# Lactobacilli Strain Mixture Alleviates Bacterial Vaginosis through Antibacterial and Antagonistic Activity in *Gardnerella vaginalis*-Infected C57BL/6 Mice

**DOI:** 10.3390/microorganisms10020471

**Published:** 2022-02-20

**Authors:** Soo-Im Choi, GaYeong Won, YongGyeong Kim, Chang-Ho Kang, Gun-Hee Kim

**Affiliations:** 1 Department of Health Functional New Materials, Duksung Women’s University, Seoul 01369, Korea; langdeveu74@duksung.ac.kr (S.-I.C.); gywon04@gmail.com (G.W.); 2 MEDIOGEN, Co., Ltd., Biovalley 1-ro, Jecheon-si 27159, Korea; yongkyung@naver.com (Y.K.); changho-kang@naver.com (C.-H.K.); 3 Department of Food and Nutrition, Duksung Women’s University, Seoul 01369, Korea

**Keywords:** lactobacilli strains mixture, *Gardnerella vaginalis*, bacterial vaginosis, epithelial exfoliation

## Abstract

The present study investigated the anti-bacterial vaginitis (BV) effects of a mixture of five lactobacilli strains (LM5), containing equal amounts of *Ligilactobacillus salivarius* MG242, *Limosilactobacillus fermentum* MG901, *Lactiplantibacillus plantarum* MG989, *Lacticaseibacillus paracasei* MG4272, and *Lacticaseibacillus rhamnosus* MG4288), in HeLa cells and *Gardnerella vaginalis* (GV)-infected BV mice. All strains produced lactic acid and hydrogen peroxide, and were resistant to nonoxynol-9. LM5 significantly inhibited GV growth by 80%, exhibited good adhesion to HeLa cells, and significantly inhibited GV adhesion to these cells. In GV-infected mice, LM5 administered orally at 5 × 10^9^ CFU/mouse significantly inhibited GV proliferation in the vaginal tract and significantly reduced myeloperoxidase activity, pro-inflammatory cytokine (TNF-α, IL-1β, and IL-6) levels, and nitric oxide levels in vaginal tissue lysates. Histopathological analysis of vaginal tissues revealed that LM5 markedly suppressed the exfoliation of vaginal epithelial cells. Overall, these results suggest that LM5 might alleviate BV by direct antibacterial and antagonistic activity in vaginal tissues of GV-infected mice.

## 1. Introduction

The vaginal environment of a healthy woman contains over 250 species of bacteria and is maintained by complex interactions or synergies between the host and microbes colonizing the vaginal mucous membrane [1,2]. Disruption of these microbial barriers can lead to a variety of urogenital diseases. Vaginitis is an inflammatory disease characterized by vaginal itching, fever, odor, and abnormal secretions from infected vaginal mucous membranes. This disease is primarily caused by infection or changes in the abnormal microbiota of the vagina [3]. Vaginitis is classified as aerobic vaginitis (AV), bacterial vaginosis (BV), vulvovaginal candidiasis, or trichomonas vaginitis, based on their etiologies [2].

BV is defined as the clinical symptoms of imbalance accompanied by an increase in vaginal pH (pH ≥ 4.5), the presence of a white adherent discharge containing exfoliated epithelial cells (clue cells), and a fishy odor [4]. BV is characterized by decreased Lactobacillus counts and excessive growth of multiple anaerobes in the vagina, such as *Gardnerella* spp., *Mycoplasma hominis*, and *Prevotella* spp. [5,6]. AV is associated with more severe inflammatory changes than BV and the presence of predominantly aerobic intestinal commensal bacteria or pathogens, including *Streptococcus agalactiae*, *Enterococcus faecalis*, *Escherichia coli*, and *Staphylococcus aureus* [7]. Despite the availability of antibiotic treatments for BV, relapse and treatment failure are common due to a failure to restore normal vaginal bacterial microbiota.

Lactic acid bacteria (LAB) such as *Lactobacillus* *crispatus*, *L. iners*, *L*. *gasseri*, and *L. jensenii* are the most prevalent in vaginal microbiota and play important roles in maintaining a healthy vaginal environment [8]. These bacteria compete with harmful bacteria in the vaginal ecosystem and act as a barrier against vaginal pathogens that cause vaginitis by producing various substances such as lactic acid, hydrogen peroxide, and bacteriocin. Lactobacilli have also been shown to reduce the risk of pregnancy-related and sexually transmitted diseases by increasing vaginal acidity [9].

Recently, probiotics have attracted increasing interest preventing and treating vaginal disorders [10]. Various types of probiotics administered orally with yogurt or as lactobacilli capsules, or by inserting lactobacilli in the form of tampons, have been studied to improve the vaginal environment [11]. Notably, a *Lactobacillus* mixture (*Lacticaseibacillus rhamnosus* GR-1 and *Limosilactobacillus fermentum* RC-14) inhibited BV recurrence [12], and a probiotic mixture (Respecta^®^; *L. rhamnosus HN001, Lactobacillus acidophilus* GLa-14, and lactoferrin RCXTM) achieved probiotic colonization in the vaginas of healthy women [13]. Although the replacement of antibiotics with probiotics to prevent vaginitis, eliminate pathogens, and improve the vaginal environment is gaining traction, studies on improving the quality of the vaginal environment using *Lactobacillus* are limited.

Our previous in vivo studies demonstrated that five lactobacilli strains (*Lacticaseibacillus paracasei* MG4272, *L. rhamnosus* MG4288, *Ligilactobacillus salivarius* MG242, *L. fermentum* MG901, and *Lactiplantibacillus plantarum* MG989) isolated from the vaginal tract of healthy Korean women have good probiotic properties, such as acid/bile salt-resistance and adhesion (auto-aggregation). Furthermore, these strains demonstrated potential antimicrobial activity against GV or *Candida albicans* [14,15,16,17,18], and a three-strain mixture (MG242, MG901, and MG989) exhibited anti-BV activity in GV-infected mice [19].

This study was undertaken to evaluate the antagonistic effects of individual strains on GV-infected HeLa cells in vitro and to determine whether a mixture of the five selected species (LM5) promotes vaginitis-improving activity in the GV infected BV mice.

## 2. Materials and Methods

### 2.1. Preparation of Lactobacilli Strains and GV

Five lactobacilli strains (*L. salivarius* MG242 (KCTC18554P), *L. fermentum* MG901 (KFCC11651P), *L. plantarum* MG989 (KFCC11650P), *L. paracasei* MG4272 (KCTC13822BP), and *L. rhamnosus* MG4288 (KCTC13823BP)), isolated from a healthy Korean woman’s vagina, were supplied by Mediogen Co., Ltd. (Jecheon, Korea). All strains were deposited in the Korean Collection for Type Cultures (KCTC, Daejeon, Korea). Each strain was activated by culture in de Man, Rogosa, and Sharpe (MRS) broth (Difco, Detroit, MI, USA) for 18 h at 37 °C [20]. The dried bacterial powder used for in vivo testing was supplied by Mediogen Co., Ltd. The lactobacilli strain mixture (LM5) was prepared by combining the five powdered strains in equal ratios.

*G. vaginalis* (KCTC5096) was obtained from the KCTC and sub-cultured in modified brain heart infusion (mBHI) broth (Difco, Detroit, MI, USA) supplemented with yeast extract (1%), maltose (0.1%), glucose (0.1%), and horse serum (10%) and cultured anaerobically using Anaerocult^®^ A (Merck, Darmstadt, Germany) in a sealed anaerobic jar.

### 2.2. Detection of Hydrogen Peroxide (H_2_O_2_) Production

The five strains were activated by culturing in 10 mL of MRS broth for 24 h at 37 °C in a bio-oxygen demand (BOD) incubator. Cultures were centrifuged and filtered to recover culture supernatants, diluted, and the pH was adjusted to 7–8. H_2_O_2_ contents in the supernatants was quantified using a commercially available kit (DoGenBio Co., Ltd., Seoul, Korea).

### 2.3. Analysis of Lactic Acid Production

Each strain was cultured in 5 mL MRS broth for 48 h, and the cultures were filtered using a 0.22 μm filter. Total lactic acid levels in the filtrates were analyzed by high-performance liquid chromatography (HPLC) using a Chiralpak^®^ MA (+) column (reverse phase-type, 4.6 × 50 mm, 5 μm, Daicel Chemicals Industries Ltd., Tokyo, Japan). The mobile phase containing 2 mM CuSO_4_ was eluted at a flow rate of 1.0 mL/min, and the sample injection volume was 10 μL. The effluent was monitored at 254 nm using a UV detector. L-(+)- and D-(−)-lactic acid (Sigma-Aldrich, St. Louis, MO, USA) were used as standard solutions.

### 2.4. Resistance to Spermicide

The five lactobacilli strains were tested for spermicide resistance by culturing them on MRS agar containing varying concentrations of nonoxynol-9 (N-9, ab143673, Abcam, Cambridge, MA, USA) as described previously with minor modifications [21]. N-9 was diluted to concentrations of 0, 6.4, 12.8, and 25.6% (*v*/*v*), respectively, with MRS broth (pH 6.5). Cultures were washed twice in phosphate-buffered saline (PBS, pH 7.1) and resuspended at 1 × 10^7^ cells/mL. The suspension (50 μL) was added to 3 mL of medium alone (control) or medium with N-9, mixed thoroughly, and incubated for 18 h in a 5% CO_2_ atmosphere at 37 °C. Growth was assessed based on visual examinations of turbidity.

### 2.5. Antibacterial Effects of Lactobacilli Strains on GV Growth

The anti-GV activities of lactobacillus strains were evaluated using cell-free culture supernatants (CFS) [19]. To prepare the CFS, the lactobacilli strains were individually cultured in MRS broth (10 mL) for 24 h at 37 °C and centrifuged at 4000× *g* for 10 min. The supernatants were filtered through a 0.2 μm membrane filter to remove debris.

GV (1 × 10^6^ CFU) was incubated in an mBHI medium containing 10% of each CFS and cultured at 37 °C for 36 h under anaerobic conditions. Viable GV numbers were then determined by diluting and plating on BHI agar containing 5% horse blood at 37 °C for 24 h under anaerobic conditions and counting CFUs.

### 2.6. The Cytotoxic Effect of Lactobacilli Strains on HeLa Cells

HeLa cells (KCLB10002) were obtained from the Korea Cell Line Bank (KCLB, Seoul, Korea). The cytotoxic effect of lactobacillus strains on HeLa cells was evaluated using an MTT assay using the CFS of each strain. Briefly, HeLa cells were cultured in RPMI 1640 supplemented with 10% fetal bovine serum (FBS) (Gibco, Grand Island, NY, USA) and incubated in a 5% CO_2_ atmosphere at 37 °C for 2 days until confluent. HeLa cell suspensions were seeded at a density of 5 × 10^4^ cells/mL in 24-well plates and incubated with CFS (100 μL) for 18 h at 37 °C in a 5% CO_2_ atmosphere. Non-treated HeLa cells were used as the controls.

### 2.7. Adhesion Ability of Lactobacilli Strains to HeLa Cells

The ability of the strains to adhere to HeLa cells was assessed [22]. HeLa cells were cultured in RPMI 1640 supplemented with 10% FBS and incubated in a 5% CO_2_ atmosphere at 37 °C for 2 days until confluence. HeLa cell suspensions were seeded at 5 × 10^4^ cells/mL in 24-well plates for 18 h at 37 °C in 5% CO_2_ and washed twice with sterile PBS.

Lactobacilli strains were prepared by culturing in MRS broth at 37 °C for 18 h. Cultures were centrifuged at 4000× *g* for 10 min at 4 °C and washed twice with PBS (pH 7.4). The pellets were resuspended in RPMI 1640 medium at a density of 2 × 10^8^ CFU/mL. Each strain suspension (500 μL) was added to each well and incubated for 1 h at 37 °C in a 10% CO_2_ atmosphere. After incubation, the cells were washed three times with sterile PBS and detached with sterile distilled water (200 μL). The cells were then diluted and plated onto MRS agar, cultured at 37 °C for 24 h under anaerobic conditions, and the number of viable lactobacilli strains was determined by counting the colonies.

### 2.8. Antagonistic Activity of Lactobacilli Strains against GV Adhesion to HeLa Cells

The antagonistic effects of each strain on GV adhesion to HeLa cells were assessed as previously described [22]. Prior to infection, HeLa cells were cultured at a density of 5 × 10^4^ cells/mL in 24-well plates, washed twice with sterile PBS, and replaced with 500 μL of fresh culture medium. GV was cultured in mBHI medium at 37 °C for 18 h under anaerobic conditions. The pellets recovered by centrifugation (4000× *g* for 10 min at 4 °C) were washed twice with PBS (pH 7.4) and resuspended in RPMI 1640 medium. Lactobacilli strains were prepared by culturing in MRS broth at 37 °C for 18 h. The recovered pellets were resuspended in RPMI 1640 medium in the same method.

Each lactobacillus strain (250 μL, 1 × 10^8^ CFU/mL) or GV (250 μL, 1 × 10^8^ CFU/mL) suspended in culture media was added to the wells and incubated for 1 h. Then, the cells were washed three times with sterile PBS and detached with sterile distilled water (200 μL). The cells were diluted and plated on Columbia Blood (CB) agar to measure the quantity of viable GV. After incubating at 37 °C for 24 h under anaerobic conditions, the bacterial colonies were counted.

### 2.9. GV-Infected BV Mice Model and LM5 Administration

C57BL/6 female mice (5 weeks, weighing 19–22 g) were obtained from Orientbio Co., (Seongnam, Korea). The mice were housed in cages under climate-controlled conditions (50% ± 10% humidity and 20–22 °C), fed standard laboratory chow (No. EEGJ36060, Furina Inc., Seongnam, Korea), and allowed water ad libitum. Animal experiments were approved by the Institutional Animal Care and Use Committee (IACUC) of Duksung Women’s University (No. 2019-011-001). All efforts were made to minimize animal suffering.

Mice were randomly allocated to the following four groups (*n* = 6/group): (1) a non-treated normal control group (NOR), (2) a BV-infected non-treated control group (CON), (3) a BV-infected LM5A (5 × 10^8^ CFU/mouse)-treated group, and (4) a BV-infected LM5B (5 × 10^9^ CFU/mouse)-treated group. To induce BV by GV infection, all mice were injected intraperitoneally with β-estradiol-3-benzoate (0.5 mg/100 mL in olive oil) 3 days before and on the day of GV inoculation, except for the NOR group. After 3 h, the mice were inoculated vaginally with GV (5 × 10^6^ CFU/20 μL) dissolved in sterile PBS, as previously described [23,24]. LM5 powder was dissolved in sterile PBS and orally administered once daily for 2 weeks following GV induction. The NOR and CON groups were treated with saline instead of the lactobacilli strains. The dosages of lactobacilli strains were determined based on our preliminary in vitro studies and a previous study by Daniel et al. [25]. The mice were sacrificed to harvest the vaginas after completing the experiment. Excised vaginal tissues were washed and stored at −80 °C until analysis.

### 2.10. GV Proliferation in Vaginal Tissues

Excised vaginal tissues were flushed with 50 μL of sterile PBS, pipetted up and down 10 times, and then recovered in a sterile Eppendorf tube. The vaginal washings were diluted by 10-fold serial dilution with PBS and then plated on GV-selective media (Thermo Scientific™, Waltham, MA, USA). GV colonies were counted, and the results were presented as CFU per 1 mL of vaginal fluid.

### 2.11. Myeloperoxidase (MPO) Activity in Vaginal Lysates

MPO activity was measured in vaginal tissue lysates. Briefly, vaginal tissues were homogenized in RIPA buffer (Sigma-Aldrich, St. Louis, MO, USA), sonicated, and centrifuged at 12,000 rpm for 20 min at 4 °C. Protein levels were measured using the Bradford method (Bio-Rad, Hercules, CA, USA). MPO activity was determined using a commercial ELISA kit (Sigma-Aldrich, St. Louis, MO, USA).

### 2.12. Pro-Inflammatory Cytokines Levels in Vaginal Tissue Lysates

The levels of inflammatory cytokines were measured in the supernatants collected from vaginal tissue lysates. Briefly, vaginal tissue lysates were homogenized in ice-cold RIPA lysis buffer containing 1% protease inhibitor cocktail and 1% phosphatase inhibitor cocktail. Lysates were centrifuged for 20 min at 4 °C, and the protein levels in the supernatants were quantified using the Bradford method. The levels of TNF-α, IL-6, IL-1β, and nitric oxide (NO) in supernatants were measured using commercial ELISA kits (R&D Systems, Minneapolis, MN, USA).

### 2.13. Histopathological Examination

The effect of LM5 on GV-infected BV mice was evaluated by analyzing the histopathological changes in vaginal tissues. Briefly, tissues were fixed in 10% formalin for at least 24 h, embedded in paraffin, sectioned at 5 μm, stained with hematoxylin and eosin (H&E), and examined under a microscope. Changes in the vaginal tissue following BV induction were evaluated by histopathological observation. The degree of exfoliation and inflammation of vaginal epithelial cells was scored from 0 to 3 as follows: 0, normal; 1, minimal; 2, moderate; 3, marked [23]. The vaginal epithelial thickness was also measured at a diameter of 30 sites randomly selected using a 40 × magnification of an optical microscope (BX51, Olympus, Japan) equipped with a camera (300MI CMOS, Aptina, CA, USA) and an image analysis system (Scope Eye, Samkyung, Korea). A board-certified toxicological pathologist performed all the histological evaluation procedures blindly.

### 2.14. Statistical Analysis

All data are expressed as the mean ± standard deviation (SD) of experiments performed in triplicate. The analysis was conducted using SPSS version 22 (IBM Corp., Armonk, NY, USA). Statistical significance was determined by one-way analysis of variance (ANOVA) followed by post hoc analysis using Dunnett’s multiple comparison tests. Statistical significance was set at *p* < 0.05.

## 3. Results

### 3.1. Lactic Acid and H_2_O_2_ Production

The production of lactic acid and H_2_O_2_ was evaluated in the culture supernatants of the five selected strains. After incubation, the pH of the culture media of MG242, MG989, MG4272, and MG4288, but not MG901, was in the range of 3.77 ± 0.019 to 3.83 ± 0.003. All strains produced H_2_O_2_, with MG901 producing the most. Lactic acid production in the culture supernatants was measured using the HPLC-UV method. All strains produced D(−)- and L(+)-lactate, and total lactic acid production was highest for MG4272, followed by MG242 strain (Table 1).

### 3.2. Nonoxynol-9 Susceptibility

As spermicides are widely used, it is essential to identify probiotic strains that can tolerate spermicidal agents. N-9 is a safe contraceptive spermicide commonly present in commercialized spermicides at a concentration of 5% [26]. N-9 is also a nonionic detergent associated with changes in commensal vaginal microflora and is particularly toxic to H_2_O_2_-producing lactobacilli strains [27,28]. We assessed whether lactobacilli strains were resistant to N-9. The maximum inhibitory concentration (MIC) of all five strains to N-9 was > 25.6%, indicating resistance (−) to N-9.

### 3.3. Antibacterial Effect of Lactobacilli Strains against GV

**Figure 1:**
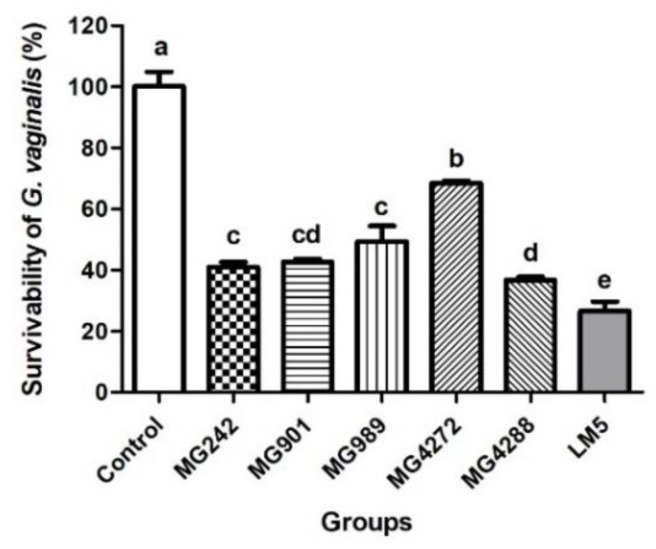
Survival rates of *Gardnerella vaginalis* (GV) alone or when treated with cell-free supernatants (CFS) of lactobacilli strains. GV was inoculated into BHI broth added with MRS broth (control) or the CFS of each of the strains and cultured for 24 h at 37 °C. LM5: mixture of five strains in the same ratio. Results are presented as mean ± SD (*n* = 3). Different letters indicate significant differences between means at *p* < 0.05 by Duncan’s multiple range test.

GV is the most dominant bacterium in the vaginal ecosystem of patients with BV and is known to be sufficient to induce the clinical symptoms of BV and related health complications [29,30]. We evaluated the in vitro antimicrobial activity of CFS obtained from five individual strains and LM5 against GV. As shown in Figure 1, the survival rate of GV significantly decreased after treatment for 24 h with all CFS. Among the single strains, MG4288 had the highest inhibition rate of 60%. In addition, the survival rate against GV was significantly higher in LM5 than in all individual strains and exhibited the highest antibacterial activity of 75% against GV.

### 3.4. Antagonistic Effect of Lactobacilli Strains on GV Adhesion to HeLa Cells

The ability of lactobacilli to adhere to vaginal epithelial cells is important for maintaining the vaginal environment by preventing pathogen adhesion, removing amine compounds, maintaining acidity, and producing antibacterial substances such as bacteriocins [31,32].

**Figure 2:**
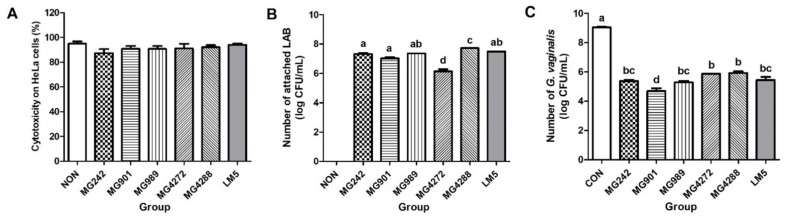
Antagonistic activity of lactobacilli strains against GV-adhesion to HeLa cells: (**A**) cytotoxic effects of cell-free culture supernatant (CFS) of lactobacilli on HeLa cells, (**B**) adhesion abilities of lactobacilli strains to HeLa cells, and (**C**) antagonistic effect of lactobacillus strains on GV adhesion to HeLa cells. NON: HeLa cells treated only with culture media, CON: HeLa cells treated only with GV without lactobacilli strains, LM5: mixture of five strains in the same ratio. Results are presented as mean ± SD (*n* = 3). Different letters indicate a significant difference between means at *p* < 0.05 by Duncan’s multiple range test.

This study confirmed the non-cytotoxic effect of the five strains and LM5 by treating HeLa cells with CFS (Figure 2A). Next, we confirmed the ability of the five strains to adhere to HeLa cells. All strains showed relatively high adhesion (>7.0 log CFU/mL), except for MG4272. MG4288 showed the highest adhesion at 7.7 log CFU/mL (Figure 2B).

In addition, the mechanism responsible for anti-BV activity was investigated by evaluating the antagonistic effect of lactobacilli strains and LM5 on GV adhesion. Each strain and LM5 inhibited GV adhesion by an average of >35% compared to GV-treated cells (CON). MG901, MG242, MG989, and LM5 reduced GV counts by >40% (Figure 2C).

### 3.5. Inhibitory Effect of LM5 on Vaginal GV Proliferation in GV-Infected BV Mice

Mice infected with GV exhibited BV-like characteristics, such as vaginal epithelial detachment, sialidase activity in vaginal fluid, mucus decomposition, and uterine infection [23]. In this study, BV was induced in mice by vaginal injection of GV (5 × 10^6^ CFU suspension), and, subsequently, infection was confirmed by the presence of intravaginal opaque mucus. The GV proliferation inhibitory activities of LM5A and LM5B on vaginal epithelial cells were evaluated by assessing the degree of colonization using the vaginal wash solution.

**Figure 3:**
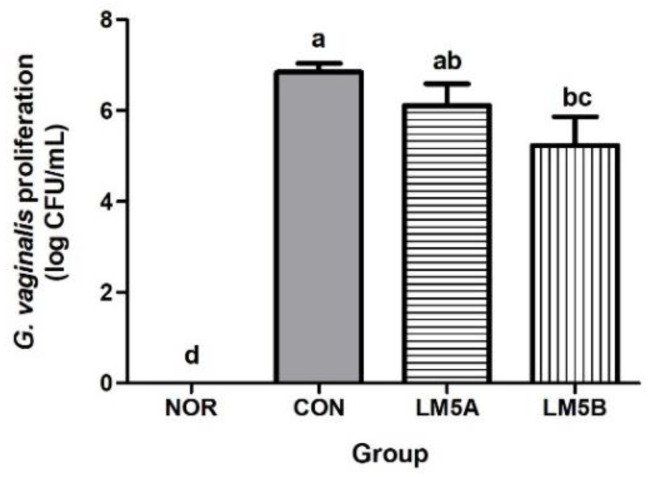
Effect of the lactobacilli strains mixture (LM5) on GV proliferation in GV-infected BV mice. Mice were fed with or without LM5A (5 × 10^8^ CFU/mouse) or LM5B (5 × 10^9^ CFU/mouse) for 2 weeks starting the day after GV inoculation. NOR: normal mice without GV infection, CON: GV infection mice without lactobacillus mixture administration. GV proliferation in mice was assessed using GV-selective agar and vaginal washes. Results are presented as mean ± SD (*n* = 6). Different letters indicate a significant difference between means at *p* < 0.05 by Duncan’s multiple range test.

The collected vaginal wash solutions were diluted and plated on a GV-selective medium. GV cells counts were markedly higher in the BV-infected CON group (6.84 ± 0.43 log CFU/mL) than in the NOR group. LM5A at 5 × 10^8^ CFU did not significantly reduce GV cell counts versus the CON group. On the other hand, LM5B at 5 × 10^9^ CFU significantly reduced GV growth to 5.15 ± 0.90 log CFU/mL (Figure 3).

### 3.6. Inhibitory Effect of LM5 on MPO Activity in Vaginal Tissue

MPO is a lysosomal protein that is highly expressed in neutrophils and plays a role in antimicrobial actions resulting from neutrophil stimulation [33]. In this study, MPO activity was measured as a biochemical index that reflects the degree of neutrophil infiltration in the vaginal tissues of GV-infected mice [34].

**Figure 4:**
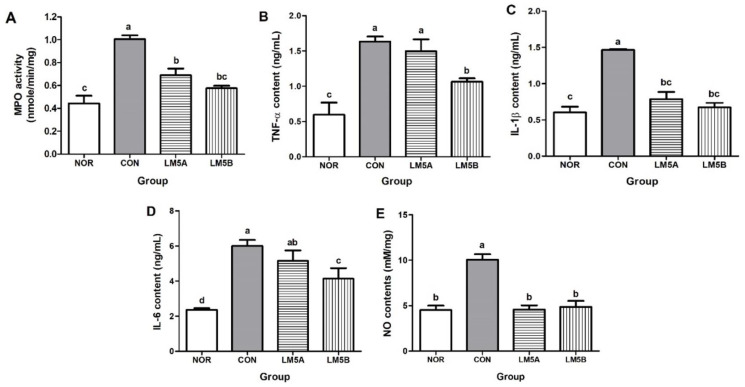
Effect of the lactobacilli strains mixture (LM5) administration on MPO activity (**A**), proinflammatory cytokines (**B**–**D**), and nitric oxide (NO) production (**E**) in GV-infected BV mice. Mice were fed with or without the LM5A (5 × 10^8^ CFU/mouse) or LM5B (5 × 10^9^ CFU/mouse) from the day after GV was inoculated for 2 weeks. MPO activity, and proinflammatory cytokine and NO levels were measured using vaginal tissue lysate. NOR: normal mice without GV infection, CON: GV infection mice without lactobacillus mixture administration. Results are presented as mean ± SD (*n* = 6). Different letters indicate a significant difference between means at *p* < 0.05 by Duncan’s multiple range test.

MPO activity was measured using vaginal tissue lysates. MPO activity in the CON group (1.01 ± 0.07 nM/min/mg) was twofold higher than in the NOR group (0.44 ± 0.13 nM/min/mg) (Figure 4A). However, MPO activities in the LM5A and B groups were significantly lower than in the CON group and were lower in the LM5B group (0.54 nm ± 0.04 nM/min/mg) than in the LM5A group (0.69 nm ± 0.10 nM/min/mg).

### 3.7. Inhibitory Effect of LM5 on Proinflammatory Cytokines Production in Vaginal Tissue

The anti-inflammatory activity of LM5 in GV-infected BV mice was investigated by measuring the levels of inflammatory cytokine in vaginal tissue lysates. TNF-α, IL-1β, IL-6, and NO levels in the CON group were significantly increased by more than twofold compared to those in the NOR group. NO levels in the LM5A and B groups were similar to those observed in the NOR group. On the other hand, TNF-α and IL-6 levels were significantly reduced in the LM5B group (Figure 4B–E).

### 3.8. Histopathological Analysis on Vaginal Tissues of GV-Infected Mice

BV is characterized by the production of white secretions containing clue cells (exfoliated epithelial cells) and Gram-positive, rod-shaped bacteria attached to the surface of vaginal tissue [35]. In this study, the extracted vaginal tissues were washed and fixed, and tissue changes were confirmed by H&E staining.

**Figure 5:**
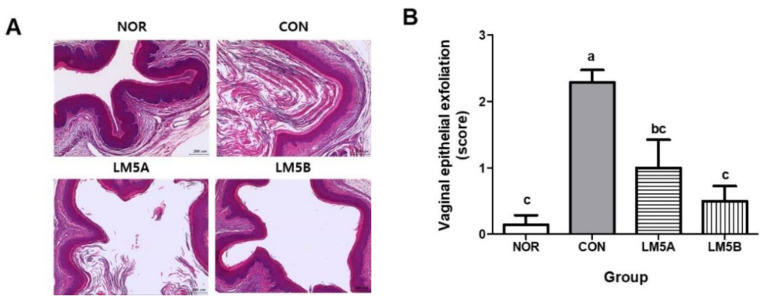
Histopathological analysis of vaginal tissues of GV-infected BV mice. (**A**) representative histopathological image of vaginal tissue, (**B**) vaginal epithelial exfoliation score. Mice were fed with or without LM5A (5 × 10^8^ CFU/mouse) or LM5B (5 × 10^9^ CFU/mouse) for 2 weeks starting the day after GV inoculation. NOR: normal mice without GV infection, CON: GV infection mice without lactobacillus mixture administration. After completing the experiment, vaginal tissues were fixed, embedded in paraffin, sectioned, and stained with hematoxylin and eosin (H&E). Results are presented as mean ± SD (*n* = 6). Different letters indicate a significant difference between means at *p* < 0.05 by Duncan’s multiple range test.

As shown in Figure 5A, epithelial cell exfoliation was clearly observed in the vaginal tissues of GV-infected mice. The CON group showed more severe epithelial cell exfoliation than the NOR group. On the other hand, the LM5A and B groups showed considerably less exfoliation. A few eosinophils were observed in the dermis of some samples, but they were within the normal range, and no group showed inflammatory cell infiltration of vaginal tissue epithelial cells. In addition, no significant intergroup differences in epithelial tissue thickness were observed.

The vaginal epithelial exfoliation score was significantly higher in the CON group (2.28 ± 0.49) than in the NOR group. The exfoliation score of the LM5B group was not significantly lower than that of the NOR group (Figure 5B).

## 4. Discussion

A lack of lactobacilli in the vaginal microflora results in the microbial environment in the vagina becoming less acidic, which favors the recurrence of BV due to the proliferation of anaerobic bacteria [36]. Although the underlying reasons for the high recurrence rate of BV are not clear [37], recurrent BV after antibiotic treatment is known to be caused by an increase in vaginal pH, increases in residual clue cells, abnormal anaerobic bacteria, and lactobacilli deficiency [10,38]. Therefore, improving the vaginal environment by maintaining vaginal acidity and inhibiting the proliferation of excessive anaerobic bacteria is important to prevent the recurrence of BV.

The mechanisms underlying the antibacterial effects of lactobacilli strains on vaginal bacterial pathogens involve hydrogen peroxide, lactic acid, bacteriocin-like molecules, and antibacterial molecules [39]. Kumherová et al. [40] reported that the H_2_O_2_ production of *L. rhamnosus* and *L. fermentum* strains were 0.7 g/L on average. However, in this study, the H_2_O_2_ production of each strain was 0.78–1.93 g/L, indicating superior production capacity. In addition, an in vitro study showed the antibacterial activity of the individual strains and mixtures against GV. LM5 significantly reduced survival rate of GV compared to that of the CFS of individual strains (Figure 1). Atassi et al. [41] reported that the survival rates of GV and *Prevotella bivia* were significantly reduced by co-treatment with culture supernatants of *L. acidophilus*, *L. jensenii*, *L. gasseri,* and *L. crispatus* isolated from healthy women. Sethi et al. [42] showed that LAB that produces hydrogen peroxide effectively inhibited GV growth. Atassi et al. [43] reported that the cooperative actions of lactic acid and hydrogen peroxide in the CFS of lactobacilli exhibited enhanced elimination of vaginitis-related pathogens. In the present study, the high lactic acid and H_2_O_2_ production abilities of the five strains corresponded to antibacterial activities against GV, and our results suggested that the lactobacilli strain mixture more effectively inhibited GV growth than individual strains.

*Lactobacillus* ingested orally can pass through the stomach and intestines and naturally migrate through the circumference of the vaginal inlet, thereby addressing vaginal microbiota imbalance [44]. The adhesion of lactobacilli to vaginal epithelial tissue is the first step toward forming a barrier that prevents undesirable microbial colonization and is the important property of probiotics intended for vaginal surface colonization [45,46]. Coudeyras et al. [47] reported the antagonistic activity of *L. rhamnosus* strain (Lcr35) against vaginal pathogens in cervical cells. Qian et al. [48] reported that three *Lactobacillus* strains (*L. delbrueckii* DM8909, *L. plantarum* ATCC14917, and *L. plantarum* ZX27) inhibited GV growth, adhesion, and biofilm formation in vitro. Similar to the results from previous studies, this study showed that all strains and the mixture adhered well to HeLa cells and exhibited potential antagonistic activity against GV in HeLa cells.

Vaginal anaerobic bacteria destroy vaginal mucosa cells by secreting various enzymes such as sialidase and proline iminopeptidase, and gray-white vaginal secretions are produced when vaginal mucosa cells are eliminated [10]. GV has excellent biofilm formation ability and promotes the adhesion of other BV-related pathogens [39]. Gilbert et al. [23] reported that GV inoculated into the vagina of mice survived for at least three days and that colonizing GV moved to the uterine horns.

In a preliminary study, we confirmed that the five-strain combination used in the present study had more significant anti-BV activity than any individual strain or a mixture of the previous three strains (Supplementary Figures S1 and S2). In this study, the BV improvement effect of a mixture of five strains (LM5) was investigated by administering two concentrations (5 × 10^8^ CFU/mouse and 5 × 10^9^ CFU/mouse) in GV-infected mice. The degree of BV induction in GV-infected mice was confirmed by assessing the survival rate of GV, inflammation, and by histological examination of vaginal tissues. In the vaginas of the BV mice, the viscosity of white vaginal fluid was high, and the number of vaginal GV colonies was significantly higher than in the NOR group. However, this GV increase in the CON group was reduced by approximately 29% in the LM5B group. Consistent with our findings, Daniel et al. [24] reported that *L. fermentum* L23 ameliorated BV by inhibiting GV growth in vaginally-infected mice. These results are considered to be due to the antibacterial and antagonistic activities of LM5 against GV, as shown in the HeLa cells in vitro.

Clinical studies have shown that GV is present on the tissue surfaces of vaginal specimens from women with BV but without apparent signs of inflammation [49,50]. Clinically, BV is regarded to be a non-inflammatory condition because it is not associated with swelling of vaginal tissues or increased neutrophils in the cervicovaginal space [51,52]. Muzny et al. [53] reported that *G. vaginalis* and *P. bivia* are abundant in women with BV inflammation but they do not induce a robust inflammatory response in vaginal epithelial cells. However, several studies have reported that pro-inflammatory cytokines such as TNF-α, IL-1β, IL-6, and IL-8 are elevated in vaginal samples of women with BV [54,55]. Wasiela [56] showed that elevated pro-inflammatory cytokine levels in the vaginas of patient with BV correlated with BV severity in certain cases.

In this study, LM5 administration significantly reduced MPO activity and pro-inflammatory cytokine and NO levels caused by GV inoculation in mice. Santos et al. [57] reported that the anti-inflammatory effects of *L. plantarum* 59 and *L. fermentum* 137 were due to inhibition of the NF-kB pathway in HeLa cells treated with GV or *Candida albicans*. Similarly, Joo et al. [22] reported that *L. johnsonii* HY7042 inhibited BV by regulating NF-κB activation and suppressing the expression of pro-inflammatory cytokines. These results suggest that LM5 administration may suppress inflammatory responses by inhibiting the production of inflammatory cytokines in GV-infected vaginas.

Normally, exfoliation removes adherent pathogens and acts as a defense mechanism against pathogens in the vagina. However, excessive exfoliation provides access to underlying tissue, facilitates the establishment of BV-related bacteria, and increases the risk of secondary infection. GV has been shown to interact with vaginal epithelial cells in culture, and thus bacterial-coated clue cells are a qualitative diagnostic feature of BV [58]. Gilbert et al. [23] established increased epithelial thickness and exfoliation of epithelial cells without an inflammatory response in GV-infected mice.

In the present study, the exfoliation of vaginal epithelial cells was clearly observed in GV-infected BV mice, and LM5 administration significantly suppressed this exfoliation. However, no significant tissue inflammation, swelling, or polymorphic nuclear cell infiltration was observed, as previously discussed. Therefore, the lactobacilli strain mixture might improve BV symptoms by reducing the exfoliation of vaginal epithelial tissue caused by GV infection. However, since there is a limitation in that the experiment was conducted using only a single strain of *G. vaginalis*, further studies to confirm the antibacterial efficacy against various pathogens may be necessary.

## 5. Conclusions

We investigated the effect of a mixture of five probiotic strains isolated from the vaginas of Korean women on BV symptoms in a GV-infected mouse model. Administration of a mixture of these five strains significantly reduced the production of vaginal inflammatory substances in the BV mice. In addition, its direct antibacterial effect and antagonistic activity against GV in vaginal tissue effectively inhibited the exfoliation of vaginal epithelial cells. Therefore, these findings suggest that LM5 has potential use as a probiotic candidate to prevent or alleviate the symptoms of BV.

## Figures and Tables

**Table 1:** Lactic acid and H_2_O_2_ production and Nonoxynol-9 (N-9) susceptibility of lactobacilli strains.

Strains	pH	H_2_O_2_ Content (g/L)	Lactic Acid Content D (−) + L (+)(g/L)	N-9 Susceptibility (+/−)
*Ligilactobacillus salivarius* MG242	3.77 ± 0.02	1.23 ± 0.00	17.44 ± 1.1	-
*Limosilactobacillusfermentum* MG901	4.32 ± 0.02	1.93 ± 0.08	11.83 ± 1.8	-
*Lactiplantibacillus plantarum* MG989	3.76 ± 0.01	0.96 ± 0.09	11.83 ± 0.9	-
*Lacticaseibacillus paracasei* MG4272	3.78 ± 0.01	0.80 ± 0.06	18.76 ± 2.4	-
*Lacticaseibacillus rhamnosus* MG4288	3.83 ± 0.01	0.78 ± 0.05	15.43 ± 1.0	-

## Data Availability

The data presented in this study are available on request from the corresponding author.

## Supplementary Materials

The following supporting information can be downloaded at: https://www.mdpi.com/article/10.3390/microorganisms10020471/s1, Figure S1: Effect of individual lactobacilli strains administration in GV-infected BV mice; Figure S2: Effect of lactobacilli strains mixture administration in GV-infection BV mice.
