# Supplementary material for: Lactobacilli Strain Mixture Alleviates Bacterial Vaginosis through Antibacterial and Antagonistic Activity in Gardnerella vaginalis-Infected C57BL/6 Mice
Source: Microorganisms. 2022 Feb 20;10(2):471. doi: 10.3390/microorganisms10020471 (PMC8880492; doi:10.3390/microorganisms10020471)
Supplement: Supplementary file 1 [file microorganisms-10-00471-s001.zip › microorganisms-1565504-supplementary.pdf]

Supplementary data

Effect of individual lactobacilli strains administration in GV-infected BV mice

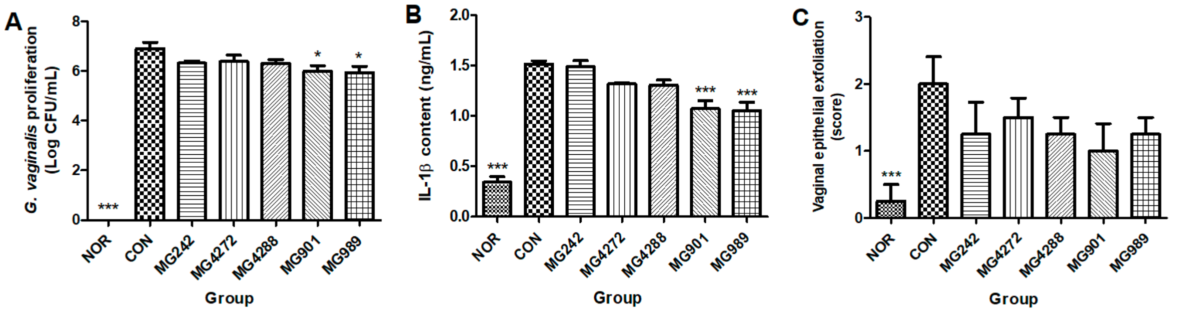

Figure S1. Effect of individual lactobacilli strains administration on GV proliferation, IL-1β production, and vagianl epithelial exfoliation in GV-infected BV mice. For the induction of BV by GV infection, mice were randomly distributed into the following seven groups (n = 6/group): (1) normal (NOR), (2) control (CON), (3) MG242, (4) MG4272, (5) MG4288, (6) MG901, and (7) MG989. All *Lactobacillus* strain groups were administered at the concentrations of 5 × 10^9 CFU/mouse orally once daily for 2 weeks from the day following GV induction.

Effect of lactobacilli strains mixture administration in GV-infection BV mice

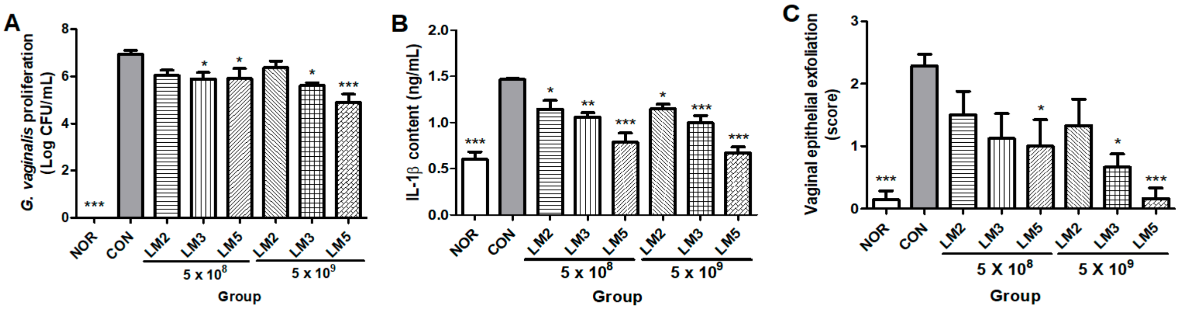

Figure S2. Effect of lactobacilli strains mixtures administration on GV proliferation, IL-1β production, and vagianl epithelial exfoliation in GV-infected BV mice. For the induction of BV by GV infection, mice were randomly distributed into the following five groups (n = 6/group): (1) normal (NOR), (2) control (CON), (3, 6) LM2 (1:1 mixture of MG4272 and MG4288), (4, 7) LM3 (1:1:1 mixture of MG242, MG901, and MG989), and (5, 8) LM5 (1:1:1:1:1 mixture of MG4272, MG4288, MG242, MG901, and MG989). All *Lactobacillus* mixtures groups were administered at two concentrations of 5 × 10^8 CFU/mouse (3, 4, and 5 groups) or 5 × 10^9 CFU/mouse (6, 7, and 8 groups) orally once daily for 2 weeks from the day following GV induction.
